# Cross-cultural adaptation into Brazilian Portuguese of an international questionnaire for the assessment of cardiopulmonary resuscitation knowledge among adolescents

**DOI:** 10.1016/j.jped.2026.101579

**Published:** 2026-07-03

**Authors:** Lucas de Moraes Soler, Gabriela Naccarati de Mello, Murilo Lima Gomes, João Marcelo Ricciardelli Riehl, Amanda Vitória Zorzi Segalla, Joelma Gonçalves Martin, Julio Cesar Garcia Alencar, Carlos Antonio Negrato

**Affiliations:** a Universidade de São Paulo, Faculdade de Medicina de Bauru, Bauru, SP, Brazil; b Universidade Estadual Paulista (UNESP), Faculdade de Medicina de Botucatu, Department of Nursing, Botucatu, SP, Brazil; c Universidade Estadual Paulista (UNESP), Faculdade de Medicina de Botucatu, Department of Pediatrics, Botucatu, SP, Brazil

**Keywords:** Cardiopulmonary resuscitation, Adolescent, Surveys and questionnaires, Translations

## Abstract

**Objective:**

To translate and cross-culturally adapt into Brazilian Portuguese an international questionnaire assessing cardiopulmonary resuscitation knowledge among adolescents.

**Methods:**

This methodological study was conducted according to internationally recommended guidelines for translation and cross-cultural adaptation. The process included forward translation, synthesis, back-translation, expert review, and pre-testing. Content validity was assessed using item-level (I-CVI) and scale-level (S-CVI) Content Validity Indexes. A pre-test evaluated clarity and comprehensibility among adolescents.

**Results:**

The questionnaire demonstrated adequate semantic, cultural, and conceptual equivalence. It comprised 35 questions, corresponding response options, and three section titles. I-CVI values ranged from 0.625 to 1.000. Seven items presented I-CVI values below 0.78 and were subsequently revised after expert review. The S-CVI was 0.92, indicating satisfactory content validity. The pre-test with 43 adolescents (mean age: 14 years) demonstrated good comprehensibility, with no significant difficulties reported.

**Conclusion:**

The questionnaire was successfully translated and cross-culturally adapted into Brazilian Portuguese, demonstrating satisfactory content validity and good comprehensibility among adolescents. Although the adapted version represents a promising tool for assessing cardiopulmonary resuscitation knowledge, further psychometric evaluation remains necessary before broader implementation.

## Introduction

Cardiac arrest (CA) is one of the leading medical emergencies worldwide, characterized by the sudden cessation of effective cardiac activity, resulting in the absence of circulation and respiration, leading to imminent death if not promptly treated [1]. It is estimated that millions of cases occur annually, a substantial proportion of which take place in out-of-hospital settings [2].

In Brazil, according to estimates from the Brazilian Society of Cardiology, approximately 200,000 cases of CA occur each year, most of them outside the hospital environment [3]. In the United States, data from the Cardiac Arrest Registry to Enhance Survival (CARES) indicate that >350,000 out-of-hospital CAs occur annually, with overall survival to hospital discharge remaining close to 10% [4].

Immediate cardiopulmonary resuscitation (CPR) performed by bystanders has been shown to double or even triple survival rates and improve neurological outcomes [5,6]. Conversely, for every minute without CPR, survival decreases by approximately 7% to 10%, underscoring the critical importance of early intervention [5]. When CPR is initiated promptly, outcomes may improve substantially, depending on response time and quality of care [4,5]. Despite the strong evidence supporting bystander CPR, intervention rates remain suboptimal, particularly in low- and middle-income countries, where cultural, structural, and educational barriers limit its dissemination [7,8]. These findings highlight the need for community-based educational strategies aimed at increasing the number of trained lay responders.

Training laypeople in CPR is recognized as one of the most effective strategies to improve survival after CA [6,9]. The American Heart Association and the European Resuscitation Council advocate for widespread Basic Life Support (BLS) training, including initiatives within school environments [5,10]. In this context, the international “Kids Save Lives” initiative, endorsed by the World Health Organization, recommends mandatory CPR training in schools starting at the age of 12 years [11].

International experiences have demonstrated that incorporating CPR training into school curricula is associated with increased rates of bystander intervention and improved survival outcomes after CA [12,13]. Studies have shown that schoolchildren from the age of 12 years are capable of acquiring basic CPR knowledge and skills and may act as multipliers of health-related practices within their communities [14,15].

In Brazil, the dissemination of CPR training remains limited. Law No 13,722/2018 established mandatory first aid training for teachers and staff in educational institutions [16]. Although this legislation represents an important advance, its implementation remains heterogeneous across educational settings and does not directly include structured CPR training programs targeting children and adolescents, who constitute a strategic population due to their potential for early learning and community impact [12].

The effectiveness of health education programs extends beyond the transmission of theoretical knowledge and may contribute to the development of competencies encompassing cognitive, psychomotor, and attitudinal domains [17]. In the context of CPR training, this includes not only understanding the principles and steps of resuscitation but also the ability and willingness to apply them appropriately in real-life situations [17,18].

As CPR training initiatives continue to expand, there is an increasing need for instruments capable of objectively evaluating their educational outcomes [17]. However, the valid and reliable assessment of CPR-related knowledge, attitudes, and practical skills remains challenging [17,18]. This challenge is particularly evident among children and adolescents, for whom there is a scarcity of specifically developed assessment tools [19]. In many cases, questionnaires originally designed for adults are applied or adapted without adequate methodological rigor, potentially compromising content validity, sensitivity to the learning process, and comparability across studies [[20], [21], [22], [23]]. Moreover, cultural and linguistic differences may influence comprehension and interpretation, highlighting the importance of culturally adapted and validated instruments [[21], [22], [23]].

Among the instruments available for assessing CPR-related educational outcomes in school settings, the questionnaire developed by Pivač et al. in Slovenia and published in 2020 was specifically designed for schoolchildren aged 12 to 15 years [24]. The instrument includes structured questions addressing key components of basic life support, such as recognition of cardiac arrest, activation of emergency medical services, chest compressions, and automated external defibrillator use, as well as items exploring attitudes toward helping behaviors and willingness to provide CPR. The original authors reported pilot testing and content validity assessment during instrument development. Its focus on adolescents, applicability to school-based educational interventions, and comprehensive assessment of CPR-related educational outcomes supported its selection for cross-cultural adaptation to the Brazilian context [24].

Thus, the aim of this study was to translate and cross-culturally adapt this questionnaire into Brazilian Portuguese for the assessment of CPR-related knowledge and attitudes among adolescents.

## Methods

### Study design

This methodological study aimed to translate and cross-culturally adapt a questionnaire designed to assess CPR knowledge among Brazilian adolescents. The study followed internationally recommended guidelines for the cross-cultural adaptation of self-report measures and for reporting studies on instrument development and validation [23].

### Original instrument and ethical considerations

The instrument selected for cross-cultural adaptation was the Questionnaire on Cardiopulmonary Resuscitation (CPR) developed by Pivač et al. for schoolchildren aged 12 to 15 years and originally designed to evaluate educational outcomes related to basic life support training in school settings [24]. The questionnaire comprises structured items organized into three sections addressing: (1) awareness and recognition of cardiopulmonary resuscitation (CPR) and automated external defibrillator (AED) concepts; (2) CPR-related knowledge; and (3) attitudes, willingness to help others, and self-confidence regarding the provision of assistance in emergency situations. The instrument was specifically developed for self-administration among adolescents in educational environments and was designed to assess both cognitive and attitudinal dimensions associated with CPR learning.

The questionnaire includes different response formats according to the construct being assessed. Knowledge-related items are presented as objective questions with predefined response options, including single-choice and multiple-choice formats, dichotomous responses, and image-based identification tasks requiring recognition of body positions, hand placement for chest compressions, and AED electrode positioning. Attitudinal items are presented as statements concerning helping behaviors, willingness to intervene, perceived responsibility toward others, and confidence in performing CPR-related actions, using a five-point Likert scale ranging from “strongly disagree” to “strongly agree”.

In the original study, knowledge items were scored dichotomously, with one point assigned to each correct response. The resulting cumulative score was used to classify participants into predefined performance categories ranging from unsatisfactory to excellent levels of CPR knowledge. Attitudinal items were analyzed separately from the knowledge domain. Rather than generating a single global score, responses to the Likert-scale items were subjected to exploratory factor analysis, which identified two latent constructs: “Attitude toward Helping Others” and “Self-confidence”. Scores for these constructs were subsequently derived from participants’ responses to the items loading on each factor, allowing assessment of psychosocial dimensions associated with bystander CPR behavior. The original authors reported satisfactory psychometric performance for these constructs and used them to evaluate changes following CPR training interventions.

Authorization for the use and cross-cultural adaptation of the instrument was formally obtained from the original author prior to study initiation.

The study was approved by the Research Ethics Committee of the Faculdade de Odontologia de Bauru, Universidade de São Paulo (FOB/USP), under protocol number 6.746.913 and CAAE 75033723.9.0000.5417, issued on April 3, 2024.

All procedures were conducted in accordance with the ethical principles established in the Declaration of Helsinki and Brazilian National Health Council Resolution No 466/2012. Written informed consent was obtained from the legal guardians (TCLE), and assent was obtained from all participating adolescents (TALE).

### Translation and cross-cultural adaptation process

**Figure 1:**
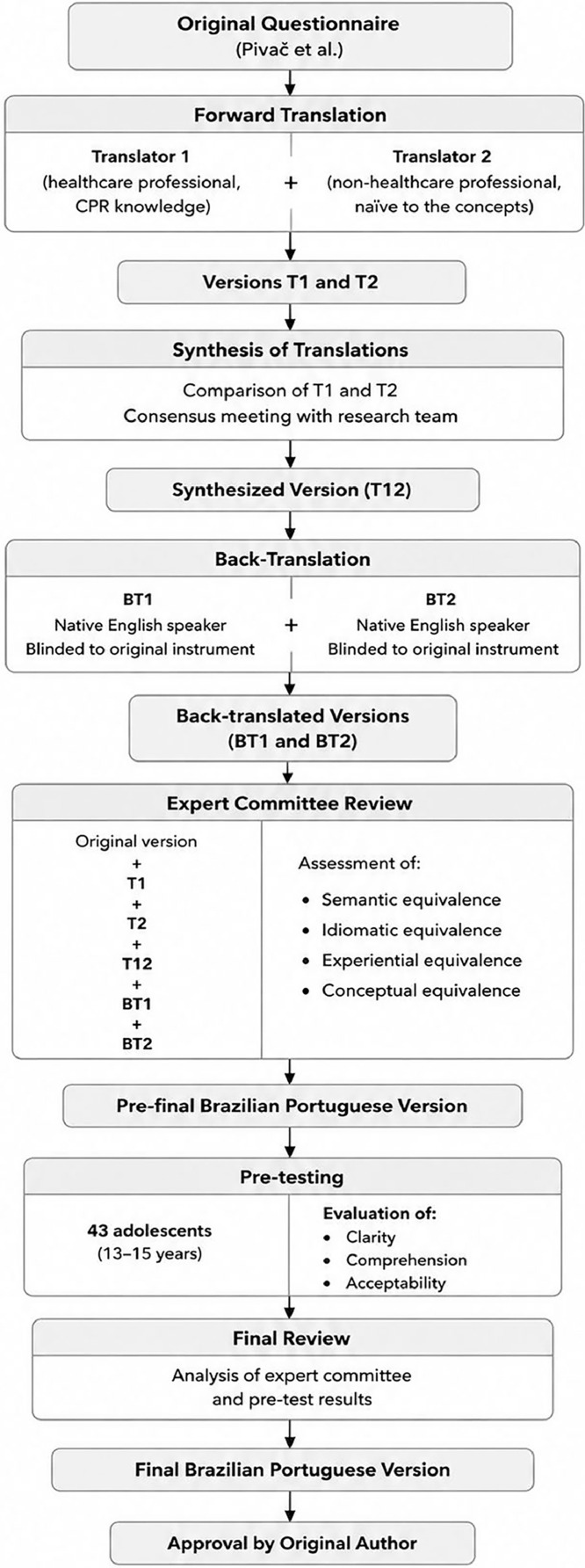
Flowchart of the translation and cross-cultural adaptation process of the CPR questionnaire into Brazilian Portuguese.

The cross-cultural adaptation process followed the six-stage methodology proposed by Beaton et al., which is widely recommended for the translation and cultural adaptation of health-related instruments [23]. The process was conducted sequentially to ensure semantic, idiomatic, experiential, and conceptual equivalence between the original questionnaire and the Brazilian Portuguese version (Figure 1).

#### Initial translation

The original English-language instrument was independently translated into Brazilian Portuguese by two bilingual translators whose native language was Portuguese. One translator had expertise in medical terminology and cardiopulmonary resuscitation (CPR), allowing preservation of the technical content of the questionnaire. The second translator had no healthcare background and was unfamiliar with the concepts explored by the instrument, thereby contributing a perspective closer to the language commonly used by the target population. Independent translations generated two preliminary versions (T1 and T2).

#### Synthesis of translations

The two translated versions were compared item by item by the translators and members of the research team. Differences in wording, grammar, terminology, and cultural appropriateness were discussed until consensus was reached. This process resulted in a synthesized Portuguese version (T12), which sought to preserve the conceptual meaning of the original instrument while ensuring linguistic clarity and cultural relevance for Brazilian adolescents.

#### Back-translation

The synthesized version (T12) was independently back-translated into English by two translators whose native language was English. Both translators were blinded to the original instrument and had no prior knowledge of the questionnaire, its objectives, or the constructs being assessed. The resulting back-translations (BT1 and BT2) were compared with the original instrument to identify potential inconsistencies, conceptual deviations, or inaccuracies introduced during translation. The purpose of this stage was not to achieve literal equivalence, but rather to verify preservation of the original meaning and content.

#### Expert committee review

The composition of the committee was consistent with recommendations for cross-cultural adaptation studies, which suggest the participation of a minimum of six experts to ensure adequate assessment of content validity [23]. The synthesized version (T12) was evaluated by an expert committee composed of eight physicians holding doctoral degrees and selected according to their complementary expertise in adult emergency medicine, pediatric emergency medicine, health education, public health, and research methodology. Collectively, the panel provided expertise in cardiopulmonary resuscitation, emergency care, health education, public health, instrument development, and methodological evaluation. The inclusion of eight specialists was intended to strengthen the evaluation process by incorporating complementary expertise across the clinical, educational, public health, and methodological domains addressed by the instrument. Professional experience among committee members ranged from 5 to 30 years (Table 1).

**Table 1:** Characteristics of the expert committee.

Specialist	Profession	Area of expertise	Years of experience
1	Physician	Emergency Medicine	15
2	Physician	Emergency Medicine	5
3	Physician	Pediatric Emergency Medicine and Methodology	25
4	Physician	Emergency Medicine	20
5	Physician	Emergency Medicine and Methodology	25
6	Physician	Pediatric Emergency Medicine	10
7	Physician	Emergency Medicine and Medical Education	20
8	Physician	Emergency Medicine and Medical Education	30

Experts assessed semantic, idiomatic, cultural, and conceptual equivalence using a four-point Likert scale. Content validity was evaluated using the Item-Level Content Validity Index (I-CVI) and the Scale-Level Content Validity Index based on the averaging method (S-CVI/Ave), as recommended by Polit and Beck [25]. The I-CVI was calculated as the proportion of experts assigning ratings of either 3 or 4 to each item, indicating that the item was considered relevant or highly relevant. The S-CVI/Ave was calculated as the average of all I-CVI values across the instrument. According to the methodological criteria proposed by Polit and Beck, I-CVI values ≥ 0.78 for panels composed of six or more experts and S-CVI/Ave values ≥0.90 were considered indicative of satisfactory content validity.

#### Standardization procedure prior to pre-test and pre-testing

According to the cross-cultural adaptation guidelines proposed by Beaton et al., pre-testing should be conducted with at least 30 individuals from the target population [23].

Because the primary objective of the pre-test phase was to assess semantic comprehension, linguistic clarity, and cultural appropriateness of the translated instrument rather than baseline CPR knowledge, participants were first provided with a standardized BLS training session. This procedure ensured a minimum conceptual framework regarding CPR, AED use, and other emergency-response concepts addressed by the questionnaire. Given the presence of technical terminology and scenario-based items, prior instruction allowed evaluation to focus on the comprehensibility and cultural adequacy of the translated content rather than on participants' familiarity with CPR-related concepts.

The training consisted of a three-hour session combining theoretical instruction, audiovisual resources, and hands-on practice with mannequins, based on current international CPR guidelines. The educational content included recognition of cardiac arrest, activation of emergency medical services, high-quality chest compressions, AED use, recovery position, and basic first-aid measures for choking and drowning situations.

After training, participants completed the questionnaire and evaluated the clarity of each item using a dichotomous scale (“clear” / “not clear”). Item clarity was calculated as the proportion of respondents rating each item as clear. Following the recommendations of Sousa and Rojjanasrirat for instrument adaptation and validation in cross-cultural health research, an agreement level of ≥ 80% was established a priori as the criterion for satisfactory comprehensibility and acceptability of each item [26].

Participants were also invited to identify items perceived as unclear and to provide comments or suggestions regarding wording, interpretation, and overall comprehensibility. Items not reaching the predefined clarity threshold would be reviewed by the research team and considered for linguistic or cultural modification. Although isolated ratings of “not clear” were observed for a small number of items, no recurring comprehension difficulties, suggestions for reformulation, or qualitative concerns were identified. All items achieved clarity indices above the established acceptability criterion, ranging from 93.0% to 100.0%. Therefore, no additional modifications were considered necessary following the pre-test phase.

#### Final version

After incorporating adjustments based on expert feedback and pre-test results, the final Brazilian Portuguese version of the questionnaire was established and approved by the original author.

### Data analysis

Content validity was assessed quantitatively using the I-CVI and S-CVI indices. Items rated as 3 or 4 by the majority of experts were considered valid. Items with I-CVI < 0.78 were revised. Additionally, qualitative analysis of expert feedback was performed to identify linguistic ambiguities and cultural inconsistencies, supporting refinement of the instrument.

## Results

### Translation and cross-cultural adaptation process

The cross-cultural adaptation process was completed according to the predefined methodological framework (Figure 1). No major difficulties were encountered during the forward translation, synthesis, back-translation, expert committee review, or pre-testing stages. Throughout the adaptation process, modifications were primarily related to linguistic refinement, cultural contextualization, and clarification of terminology to ensure semantic, idiomatic, experiential, and conceptual equivalence between the original instrument and the Brazilian Portuguese version.

Several items underwent wording adjustments to improve comprehensibility and cultural appropriateness for Brazilian adolescents while preserving the conceptual meaning of the original questionnaire. A complete comparison of all versions generated during the adaptation process, including the original instrument, forward translations (T1 and T2), synthesized version (T12) and back-translations (BT1 and BT2), is presented in Supplementary Table S1.

### Expert committee evaluation and content validity

The pre-final version (T12) was evaluated by the expert committee regarding semantic, idiomatic, cultural, and conceptual equivalence.

I-CVI values ranged from 0.625 to 1.000. Seven questionnaire items presented I-CVI values below 0.78 and were subsequently revised based on the experts’ recommendations. These revisions focused on improving linguistic clarity, cultural adequacy, and conceptual precision while maintaining fidelity to the original instrument. The overall content validity of the instrument was considered satisfactory, with a Scale-Level Content Validity Index based on the averaging method (S-CVI/Ave) of 0.92.

The complete distribution of expert ratings for all questionnaire items, including individual item scores and corresponding I-CVI values, is provided in Supplementary Table S2.

### Participant characteristics and pre-test results

The pre-test was conducted with 43 adolescents aged 13–15 years (Table 2). Participant demographic characteristics are presented in Table 2.

**Table 2:** Characteristics of the adolescents included in the pre-test.

Variable	Value
Female, n (%)	26 (60%)
Number of participants	43
Age, years (mean)	14
Age range, years	13–15
Age, n (%)	
13 years	5 (12%)
14 years	29 (67%)
15 years	9 (21%)
Educational level	Elementary school

All questionnaire items exceeded the predefined comprehensibility threshold of 80%. Item-level clarity indices ranged from 93.0% to 100.0%, indicating a high degree of understanding among participants.

Although isolated “not clear” responses were observed for a small number of items, no recurring comprehension difficulties, suggestions for reformulation, or qualitative concerns were identified during the pre-test. Consequently, no additional modifications were considered necessary following this phase.

The complete item-by-item clarity assessment, including the percentage of participants rating each item as “clear” or “not clear,” is presented in Supplementary Table S3.

Following completion of the adaptation process, the final Brazilian Portuguese version of the questionnaire was submitted to the original author for review and approval. The author reviewed and approved the final adapted version after completion of the translation, expert committee evaluation, and pre-testing stages. The final Brazilian Portuguese version of the instrument is available in Supplementary Material S4.

## Discussion

This study successfully achieved its primary objective of translating and cross-culturally adapting the questionnaire developed by Pivač et al. [24] for use among Brazilian adolescents. Following internationally recognized recommendations for cross-cultural adaptation, the process resulted in a Brazilian Portuguese version that preserved the semantic, idiomatic, cultural, and conceptual equivalence of the original instrument while demonstrating satisfactory content validity. These findings support the use of the adapted questionnaire as an appropriate tool for assessing CPR-related knowledge and attitudes among Brazilian adolescents [23,26].

The content validity observed in the present study (S-CVI/Ave = 0.92) indicates a high level of agreement among experts and is consistent with findings from previous methodological studies involving the adaptation of health-related instruments. According to Polit and Beck, S-CVI values above 0.90 are considered indicative of excellent content validity, reinforcing the robustness of the adapted version [25]. A comparable contribution to the field was provided by Gradvohl et al., who developed and validated a questionnaire specifically designed to assess resuscitation-related knowledge and attitudes among adolescents [20]. Similar to the present investigation, their study addressed the need for age-appropriate instruments capable of evaluating educational outcomes associated with school-based CPR training. However, important methodological distinctions should be acknowledged. Whereas Gradvohl et al. focused on the development and psychometric validation of a novel instrument within its original cultural context, the present study was designed to translate and cross-culturally adapt an existing questionnaire previously developed for adolescents. Consequently, the authors’ objective was not to establish the psychometric properties of a newly developed instrument, but rather to ensure semantic, idiomatic, cultural, and conceptual equivalence while preserving the theoretical constructs underlying the original version.

Despite these methodological differences, both investigations underscore the importance of rigorously developed and population-specific assessment tools for strengthening the evaluation of CPR educational interventions among adolescents [20].

Although the overall S-CVI/Ave was excellent, seven items initially presented I-CVI values below the recommended threshold and therefore required revision. Rather than representing a weakness of the adaptation process, these findings highlight the importance of expert evaluation in identifying linguistic and cultural nuances that could influence item interpretation. The iterative revision process allowed these issues to be addressed while preserving the conceptual meaning of the original questionnaire, ultimately strengthening the quality of the final version [23,25,26].

The modifications performed throughout the adaptation process primarily involved adjustments in terminology, wording, and cultural references to ensure comprehension and contextual appropriateness for Brazilian adolescents. This finding reinforces the premise that linguistic equivalence alone is insufficient to guarantee the validity of an instrument across different cultural settings. As emphasized by Beaton et al. and Guillemin et al., successful cross-cultural adaptation requires preserving the conceptual intent of each item while ensuring that the target population interprets the content as originally intended [22,23]. The structured methodology adopted in this study contributed to maintaining the integrity of the original constructs while improving cultural relevance and comprehensibility.

The selection of the questionnaire developed by Pivač et al. [24] was particularly appropriate because the original instrument was specifically designed to evaluate CPR-related knowledge and attitudes among school-aged adolescents following educational interventions in basic life support. Unlike instruments originally developed for adults and subsequently applied to younger populations, the questionnaire was conceived within an educational context targeting adolescents. Consequently, the adaptation process required predominantly linguistic and cultural adjustments rather than substantial conceptual modifications, supporting the preservation of the theoretical constructs assessed by the original instrument.

The findings of this study are aligned with international evidence emphasizing the importance of validated instruments in the evaluation of CPR training programs, particularly in school settings [11,13,21,24,27]. The incorporation of CPR education into school curricula has been strongly recommended by organizations such as the World Health Organization and the European Resuscitation Council, which advocate the implementation of regular CPR training beginning in adolescence [5,10,11]. In this context, the availability of reliable and culturally adapted assessment tools is essential for measuring educational outcomes, monitoring program effectiveness, and supporting the development of evidence-based educational strategies.

Recent studies have demonstrated that school-based CPR training significantly improves knowledge acquisition and practical skills among adolescents, contributing to increased willingness to intervene in emergency situations and potentially improving survival after out-of-hospital cardiac arrest [12,14]. Nevertheless, despite growing international support for CPR education in schools, important barriers to implementation remain, particularly in low- and middle-income countries [8]. In Brazil, although Law No 13,722/2018 expanded first-aid training requirements for school personnel, structured CPR education programs directed at students remain limited and heterogeneous. Additional challenges may include variability in school infrastructure, limited availability of trained instructors and educational resources, competing curricular priorities, and insufficient public awareness regarding the importance of bystander CPR [7,16]. These challenges reinforce the need for validated instruments capable of systematically evaluating educational initiatives and generating evidence to support future implementation efforts.

The results of the pre-test further support the adequacy of the adapted instrument. Adolescents demonstrated a high level of comprehension and reported no significant difficulties in understanding the questionnaire items. This finding is particularly relevant because clarity and accessibility of language are fundamental characteristics of self-reported measures intended for younger populations [26]. Ensuring that questionnaire items are easily understood reduces the likelihood of response bias and contributes to the reliability and accuracy of the collected data.

From an educational perspective, it is important to recognize that the present instrument evaluates only specific dimensions of CPR competence. The questionnaire primarily assesses cognitive aspects related to CPR knowledge and selected attitudinal components. However, contemporary educational frameworks emphasize that professional and layperson competence encompasses multiple domains, including knowledge, psychomotor skills, attitudes, and behavioral readiness [18,19]. Therefore, although the adapted questionnaire represents an important tool for evaluating learning outcomes, it should not be interpreted as a comprehensive measure of overall CPR competence.

Current evidence suggests that knowledge acquisition alone may not be sufficient to ensure effective action during a real cardiac arrest event. Factors such as self-efficacy, confidence, willingness to intervene, and psychomotor performance play critical roles in determining bystander response [17]. Consequently, future studies evaluating CPR educational programs among adolescents should consider combining the present questionnaire with complementary assessment methods, such as practical skill checklists, simulation-based performance assessments, and self-efficacy measures, to obtain a more comprehensive evaluation of CPR-related competencies [17,19,27].

This study has some limitations that should be acknowledged. First, the research focused on translation, cross-cultural adaptation, and content validity, without evaluating additional psychometric properties such as internal consistency, test-retest reliability, responsiveness, and construct validity. These properties are essential for establishing the overall measurement quality of the instrument and should be investigated in future studies. Second, the pre-test was conducted in a single educational setting, which may limit the generalizability of the findings to other regions and educational contexts in Brazil. Third, although the expert committee included professionals with extensive experience in emergency medicine, medical education, and research methodology, all members were physicians. The inclusion of experts from other disciplines might have provided complementary perspectives regarding educational and measurement-related aspects of the instrument. Nevertheless, the expertise represented within the committee was considered appropriate for evaluating the semantic, idiomatic, cultural, and conceptual equivalences required during the cross-cultural adaptation process.

Despite these limitations, the study presents important strengths. The use of a rigorous methodological framework based on internationally recognized recommendations ensured transparency and reliability throughout the adaptation process [22,23]. The participation of an expert committee with extensive experience in emergency medicine and medical education contributed to the quality of the content validation. Furthermore, the inclusion of adolescents during the pre-test phase ensured that the final version was evaluated within its target population, enhancing its practical applicability. Taken together, these strengths support the use of the adapted questionnaire as a valuable tool for future research and educational initiatives aimed at promoting CPR training among Brazilian adolescents.

The questionnaire was successfully translated and cross-culturally adapted into Brazilian Portuguese through a rigorous methodological process. The adapted version demonstrated adequate semantic, idiomatic, cultural, and conceptual equivalence, satisfactory content validity, and good comprehensibility among Brazilian adolescents.

The present findings provide initial evidence supporting the suitability of the instrument for use in the Brazilian context. However, further studies evaluating reliability, construct validity, and other psychometric properties are required before broader implementation in research and educational programs. Such investigations will contribute to establishing the instrument as a robust tool for assessing CPR-related knowledge among Brazilian adolescents.

## Funding

This research did not receive any specific grant from funding agencies in the public, commercial, or not-for-profit sectors.

## Data availability

The data that support the findings of this study are available from the corresponding author.

## Conflicts of interest

The authors declare no conflicts of interest.

## Appendix Supplementary materials

Image, application 1

Image, application 2

Image, application 3

Image, application 4

## References

[1] Merchant R.M., Topjian A.A., Panchal A.R., Cheng A., Aziz K., Berg K.M. (2020). Adult basic and advanced life support, pediatric basic and advanced life support, neonatal life support, resuscitation education science, and systems of care writing groups. Part 1: executive summary: 2020 American heart association guidelines for cardiopulmonary resuscitation and emergency cardiovascular care. Circulation.

[2] Berdowski J., Berg R.A., Tijssen J.G., Koster R.W. (2010). Global incidences of out-of-hospital cardiac arrest and survival rates: systematic review of 67 prospective studies. Resuscitation.

[3] Bernoche C., Timerman S., Polastri T.F., Giannetti N.S., Siqueira A.W., Piscopo A. (2019). Atualização da Diretriz de Ressuscitação Cardiopulmonar e Cuidados Cardiovasculares de Emergência da Sociedade Brasileira de Cardiologia - 2019. Arq Bras Cardiol.

[4] Buaprasert P., Al-Araji R., Rajdev M., Vellano K., J Carr M., McNally B. (2024). The past, present, and future of the cardiac arrest registry to enhance survival (CARES). Resusc Plus.

[5] Perkins G.D., Graesner J.T., Semeraro F., Olasveengen T., Soar J., Lott C. (2021). European resuscitation council guideline collaborators. European resuscitation council guidelines 2021: executive summary. Resuscitation.

[6] Berg K.M., Bray J.E., Ng K.C., Liley H.G., Greif R., Carlson J.N. (2023). 2023 international consensus on cardiopulmonary resuscitation and emergency cardiovascular care science with treatment recommendations: summary from the basic life support; advanced life support; pediatric life support; neonatal life support; education, implementation, and teams; and first aid task forces. Circulation.

[7] Maia S.R., Lemos A.M., Frutuoso M.S., Júnior C.W. (2020). Knowledge of laity about cardiopulmonary resuscitation in adults in Brazil. Braz J Dev.

[8] Kiguchi T., Okubo M., Nishiyama C., Maconochie I., Ong M.E., Kern K.B. (2020). Out-of-hospital cardiac arrest across the world: first report from the international liaison committee on resuscitation (ILCOR). Resuscitation.

[9] Semeraro F., Greif R., Böttiger B.W., Burkart R., Cimpoesu D., Georgiou M. (2021). European resuscitation council guidelines 2021: systems saving lives. Resuscitation.

[10] Böttiger B.W., Van Aken H. (2015). Kids save lives–training school children in cardiopulmonary resuscitation worldwide is now endorsed by the world health organization (WHO). Resuscitation.

[11] World Health Organization (2015).

[12] Bohn A., Van Aken H.K., Möllhoff T., Wienzek H., Kimmeyer P., Wild E. (2012). Teaching resuscitation in schools: annual tuition by trained teachers is effective starting at age 10. A four-year prospective cohort study. Resuscitation.

[13] Böttiger B.W., Van Aken H. (2015). Training children in cardiopulmonary resuscitation worldwide. Lancet.

[14] Isbye D.L., Rasmussen L.S., Ringsted C., Lippert F.K. (2007). Disseminating cardiopulmonary resuscitation training by distributing 35,000 personal manikins among school children. Circulation.

[15] Zenani N.E., Bello B., Molekodi M., Useh U. (2022). Effectiveness of school-based CPR training among adolescents to enhance knowledge and skills in CPR: a systematic review. Curationis.

[16] Brazil. Law No. 13,722, October 4, 2018. Mandatory training in basic first aid in educational institutions. Official gazette of the union; 2018.

[17] Monteiro M.L., Ferraz A.I., Rodrigues F.M. (2021). Assessment of knowledge and self-efficacy before and after teaching basic life support to schoolchildren. Rev Paul Pediatr.

[18] Frank J.R., Snell L.S., Cate O.T., Holmboe E.S., Carraccio C., Swing S.R. (2010). Competency-based medical education: theory to practice. Med Teach.

[19] Miller G.E. (1990). The assessment of clinical skills/competence/performance. Acad Med.

[20] Gradvohl E., Lukács Á.J., Takács J., Fritúz G., Falus A., Feith H.J. (2023). Development and validation of the questionnaire on resuscitation-related knowledge and attitude for adolescents. Eval Program Plann.

[21] Greif R., Lockey A.S., Conaghan P., Lippert A., De Vries W., Monsieurs K.G. (2015). Education and implementation of resuscitation section collaborators; collaborators. European resuscitation council guidelines for resuscitation 2015: section 10. Education and implementation of resuscitation. Resuscitation.

[22] Guillemin F., Bombardier C., Beaton D. (1993). Cross-cultural adaptation of health-related quality of life measures: literature review and proposed guidelines. J Clin Epidemiol.

[23] Beaton D.E., Bombardier C., Guillemin F., Ferraz M.B. (2000). Guidelines for the process of cross-cultural adaptation of self-report measures. Spine (Phila Pa 1976).

[24] Pivač S., Gradišek P., Skela-Savič B. (2020). The impact of cardiopulmonary resuscitation (CPR) training on schoolchildren and their CPR knowledge, attitudes toward CPR, and willingness to help others and to perform CPR: mixed methods research design. BMC Public Health.

[25] Polit D.F., Beck C.T. (2006). The content validity index: are you sure you know what's being reported? Critique and recommendations. Res Nurs Health.

[26] Sousa V.D., Rojjanasrirat W. (2011). Translation, adaptation and validation of instruments or scales for use in cross-cultural health care research: a clear and user-friendly guideline. J Eval Clin Pr.

[27] Greif R., Bhanji F., Bigham B.L., Bray J., Breckwoldt J., Cheng A. (2020). Education, implementation, and teams: 2020 international consensus on cardiopulmonary resuscitation and emergency cardiovascular care science with treatment recommendations. Resuscitation.

