# Supplementary material for: Cross-cultural adaptation into Brazilian Portuguese of an international questionnaire for the assessment of cardiopulmonary resuscitation knowledge among adolescents
Source: J Pediatr (Rio J). 2026 Jul 3;102(5):101579. doi: 10.1016/j.jped.2026.101579 (PMC13355626; doi:10.1016/j.jped.2026.101579)
Supplement: Supplementary file 4 [file mmc4.docx]

### Supplementary Material S4- Final Brazilian Portuguese Version of the CPR Questionnaire

This supplementary material presents the final Brazilian Portuguese version of the questionnaire after the cross-cultural adaptation process.

Questionário sobre Reanimação Cardiopulmonar (RCP)

PARTE 1: Reanimação Cardiopulmonar (RCP) e Desfibrilador Externo Automático (DEA)

1. Você sabe qual o número do telefone para emergências médicas no Brasil?

a) Sim

b) Não

Se souber, escreva aqui: __________

1. O que você deve informar ao atendente do SAMU 192 sobre a pessoa que você encontrou e que não apresenta sinais de vida? (mais de uma resposta é possível)

a) Minha localização

b) Não preciso informar meu local se o SAMU 192 conseguir rastrear minha localização

c) O que aconteceu

d) Quantas pessoas precisam de ajuda

e) Meu número de telefone

f) Outro: ______________

1. O que você faria se alguém desmaiasse na sua frente e não apresentasse qualquer sinal de vida? (mais de uma resposta é possível)

a) Eu me afastaria, não teria coragem de me aproximar da pessoa

b) Eu ligaria para o número de emergência

c) Eu verificaria se a pessoa está consciente (se reage)

d) Se a pessoa não estiver reagindo, eu começaria a Reanimação Cardiopulmonar (RCP)

e) Se a pessoa não estiver reagindo, eu a colocaria na posição para pessoas inconscientes

f) Outro: ______________

1. Qual a posição certa para pessoas inconscientes (como elas devem ser posicionadas)?


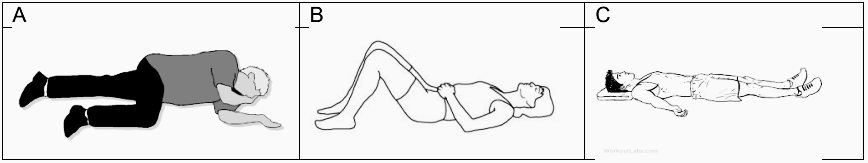


a) A figura A é a posição certa

b) A figura B é a posição certa

c) A figura C é a posição certa

1. O que é um DEA (Desfibrilador Externo Automático)? (marque somente uma resposta)

a) Um aparelho eletrônico que verifica se o coração de uma pessoa está batendo

b) Um aparelho eletrônico que verifica a respiração de uma pessoa

c) Um aparelho eletrônico que verifica e restabelece a função cardíaca

1. Sua escola tem um aparelho DEA (Desfibrilador Externo Automático)?

a) Sim

b) Não

c) Não sei

1. Você sabe em quais lugares um aparelho DEA (Desfibrilador Externo Automático) pode ser encontrado?

a) Sim

b) Não

1. Se a resposta anterior foi "sim", cite alguns lugares onde é possível encontrar um DEA (Desfibrilador Externo Automático):

__________________________________________________________________

PARTE 2: Conhecimentos sobre Reanimação Cardiopulmonar (RCP)

1. O que você deve fazer primeiro quando uma pessoa não apresenta sinais de vida ou não reage? (marque somente uma resposta)

a) Localize as possíveis vítimas e garanta que o local é seguro

b) Ligue para 192 e aguarde o SAMU

c) Garanta que o local é seguro, localize as possíveis vítimas e ligue para 192

1. Como verificar se uma pessoa está respirando? (marque somente uma resposta)

a) Incline a cabeça da pessoa para trás, ouça os sons de respiração pela boca e observe se o tórax sobe e desce

b) Incline a cabeça da pessoa para trás e observe se o tórax sobe e desce

c) Segure a pessoa pelos ombros e sacuda levemente

1. Qual deve ser a profundidade das compressões torácicas? (marque somente uma resposta)

a) 5 cm

b) 7 cm

c) 3 cm

1. Quantas compressões torácicas devem ser realizadas por minuto? (marque somente uma resposta)

a) 60 – 80 compressões por minuto

b) 130 – 150 compressões por minuto

c) 100 – 120 compressões por minuto

1. A imagem do tórax tem diferentes posições marcadas; indique o número que corresponde à posição onde as mãos devem ser colocadas para realizar as compressões torácicas:


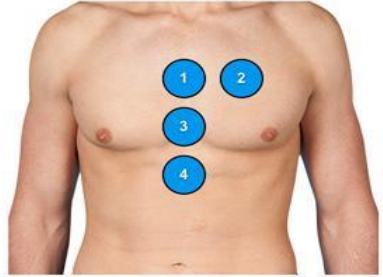


Resposta: _________________________________

1. A imagem do tórax tem diferentes posições marcadas; indique os números que correspondem às posições onde os eletrodos do DEA devem ser colocados:


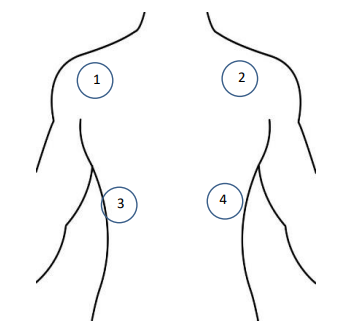


Resposta: _________________________________

1. Qual a sequência correta de ações que você deve fazer ao utilizar um DEA (Desfibrilador Externo Automático)? (mais de uma resposta é possível)

a) Ligue para 192, permaneça no local e aguarde o SAMU 192.

b) Verifique se a pessoa está reagindo e respirando, depois ligue o DEA e siga as instruções.

c) Ligue para 192 para receber orientações sobre o uso do DEA.

1. Como você começaria a reanimação de uma vítima de afogamento? (marque somente uma resposta)

a) Verificaria a respiração, iniciaria as compressões torácicas e realizaria duas respirações boca a boca.

b) Verificaria a respiração, faria cinco respirações boca a boca iniciais e, em seguida, faria as compressões torácicas.

c) Verificaria a respiração e iniciaria as compressões torácicas.

PARTE 3: Atitudes em relação a uma pessoa que precisa de ajuda*

*Esta parte tem como objetivo avaliar sua atitude em relação a uma pessoa que precisa de ajuda e como você reagiria se encontrasse uma vítima.

1. Quando alguém desmaiar na minha frente, eu ajudarei.

a) Discordo totalmente

b) Discordo

c) Não concordo, nem discordo

d) Concordo

e) Concordo totalmente

1. Quando alguém que gosto desmaiar na minha frente, eu ajudarei.

a) Discordo totalmente

b) Discordo

c) Não concordo, nem discordo

d) Concordo

e) Concordo totalmente

1. Estou disposto a ajudar quem precisa, porque eu gostaria de ser ajudado em uma emergência médica.

a) Discordo totalmente

b) Discordo

c) Não concordo, nem discordo

d) Concordo

e) Concordo totalmente

1. Eu ajudo porque consigo reconhecer quando alguém não apresenta sinais de vida.

a) Discordo totalmente

b) Discordo

c) Não concordo, nem discordo

d) Concordo

e) Concordo totalmente

1. Gosto de ajudar os outros quando precisam.

a) Discordo totalmente

b) Discordo

c) Não concordo, nem discordo

d) Concordo

e) Concordo totalmente

1. Percebo quando alguém perto de mim precisa de ajuda em uma situação de emergência médica.

a) Discordo totalmente

b) Discordo

c) Não concordo, nem discordo

d) Concordo

e) Concordo totalmente

1. Quando percebo que alguém precisa de ajuda, pergunto a mim mesmo como posso ajudar.

a) Discordo totalmente

b) Discordo

c) Não concordo, nem discordo

d) Concordo

e) Concordo totalmente

1. Quando alguém desmaiar na minha frente, eu pedirei ajuda imediatamente.

a) Discordo totalmente

b) Discordo

c) Não concordo, nem discordo

d) Concordo

e) Concordo totalmente

1. Quando eu notar um grupo de pessoas apenas observando uma vítima sem sinais de vida, eu começarei a ajudar imediatamente.

a) Discordo totalmente

b) Discordo

c) Não concordo, nem discordo

d) Concordo

e) Concordo totalmente

1. Sei que, ao ajudar alguém sem sinais de vida, eu posso salvar essa pessoa.

a) Discordo totalmente

b) Discordo

c) Não concordo, nem discordo

d) Concordo

e) Concordo totalmente

1. Quando alguém desmaiar na minha frente sem sinais de vida, eu iniciarei a Reanimação Cardiopulmonar (RCP).

a) Discordo totalmente

b) Discordo

c) Não concordo, nem discordo

d) Concordo

e) Concordo totalmente

1. Quando eu perceber uma pessoa que não está se mexendo ou não apresenta sinais de vida, eu começarei o atendimento imediatamente, porque não tenho medo de machucá-la.

a) Discordo totalmente

b) Discordo

c) Não concordo, nem discordo

d) Concordo

e) Concordo totalmente

1. Quando eu perceber uma pessoa que não está se mexendo ou não apresenta sinais de vida, eu iniciarei a Reanimação Cardiopulmonar (RCP) imediatamente, porque confio em mim.

a) Discordo totalmente

b) Discordo

c) Não concordo, nem discordo

d) Concordo

e) Concordo totalmente

1. Quando alguém que eu gosto está em situação de emergência médica, eu não hesito e ajudo imediatamente.

a) Discordo totalmente

b) Discordo

c) Não concordo, nem discordo

d) Concordo

e) Concordo totalmente

1. Eu fico triste quando alguém que eu gosto está em situação de emergência médica.

a) Discordo totalmente

b) Discordo

c) Não concordo, nem discordo

d) Concordo

e) Concordo totalmente

1. Acho que uma das melhores coisas é poder ajudar alguém.

a) Discordo totalmente

b) Discordo

c) Não concordo, nem discordo

d) Concordo

e) Concordo totalmente

1. Fico feliz em poder ajudar alguém.

a) Discordo totalmente

b) Discordo

c) Não concordo, nem discordo

d) Concordo

e) Concordo totalmente

1. Eu teria coragem de realizar Reanimação Cardiopulmonar (RCP) antes de receber o treinamento.

a) Discordo totalmente

b) Discordo

c) Não concordo, nem discordo

d) Concordo

e) Concordo totalmente

1. Eu teria coragem de realizar Reanimação Cardiopulmonar (RCP) depois de receber o treinamento.

a) Discordo totalmente

b) Discordo

c) Não concordo, nem discordo

d) Concordo

e) Concordo totalmente
