# Supplementary material for: Cross-cultural adaptation into Brazilian Portuguese of an international questionnaire for the assessment of cardiopulmonary resuscitation knowledge among adolescents
Source: J Pediatr (Rio J). 2026 Jul 3;102(5):101579. doi: 10.1016/j.jped.2026.101579 (PMC13355626; doi:10.1016/j.jped.2026.101579)
Supplement: Supplementary file 1 [file mmc1.pdf]

ORIGINAL	TRADUÇÃO - Tradutora T1	TRADUÇÃO - Tradutora T2	SÍNTESE T12 (Ana Zuleika Pinheiro Machado, Alessandra Cani Gonzales Harmel)	RETROTRADUÇÃO - BT1 (Robert Dinham - naive to outcome measurement)	RETROTRADUÇÃO - BT2 (Mark Edward Murray - naive to outcome measurement)
**SET 2: CPR AND AUTOMATED EXTERNAL DEFIBRILLATOR (AED)**	**CONJUNTO 2: RESSUSCITAÇÃO CARDIORESPIRATÓRIA (RCP) E DESFIBRILADOR EXTERNO AUTOMÁTICO (DEA)**	**PARTE 2: RCP E DESFRIBILADOR EXTERNO AUTOMÁTICO (DEA)**	**Parte 2: REANIMAÇÃO CARDIOPULMONAR (RCP) E DESFIBRILADOR EXTERNO AUTOMÁTICO (DEA)**	**PART 2: CARDIOPULMONARY RESUSCITATION (CPR) AND THE AUTOMATED EXTERNAL DEFIBRILLATOR (AED)**	**PART 2: CARDIOPULMONARY RESUSCITATION (CPR) AND AUTOMATED EXTERNAL DE**
1 Do you know the phone number for medical emergencies in Slovenia?	01 Você sabe qual o número de telefone para emergências médicas na Eslovênia?	1. Você sabe o número de telefone para chamar em caso de emergência médica na Eslovênia?	01 Você sabe qual o número de telefone para emergências médicas no Brasil?	01 Do you know the telephone number for medical emergencies in Brazil?	01 Do you know the telephone number for medical emergencies in Brazil?
1. Yes	1. Sim	1. Sim	1. Sim	1. Yes.	1. Yes, I do
2. No	2. Não	2. Não	2. Não	2. No.	2. No, I don't
If you know the number, please write it here:	Se souber, escreva aqui: __________	Se souber o número, escreva-o aqui: [linha para preenchimento]	Se souber, escreva aqui: __________	If you know it, write it here: _______	If you know it, write it down here: __________
2 What must you tell healthcare dispatchers about the person you found who is not showing signs of life? (more than one answer is possible)	02. O que você deve informar ao atendente da central de emergências sobre a pessoa que você encontrou e que não apresenta sinais de vida? (mais de uma resposta é possível)	2. O que você deve dizer aos atendentes da central de saúde sobre a pessoa que você encontrou e que não est	02. O que você deve informar ao atendente da central de emergências sobre a pessoa que você encontrou e que não mostrar si	2. What should you tell the emergency call center dispatcher about the person you found who is without signs of life? (more th	02. What should you say to the emergency call center operator about the person you found wh
1.) My location	1.) Minha localização	1. Minha localização	1. Minha localização	1. My location.	1. My location
2.) My location is not important because I'm using a smartphone	2.) A localização não é importante, pois estou usando um smartphone	2. Minha localização não é importante porque estou usando um smartphone	2. Minha localização não é importante porque estou usando um telefone	2. My location is not important because I'm using a smartphone.	2. My location is not important because I am using a smartphone
3.) What happened	3.) O que aconteceu	3. O que ocorreu	3. O que aconteceu	3. What happened.	3. What has happened
4.) How many people need assistance	4.) Quantas pessoas precisam de ajuda	4.) Quantas pessoas precisam de ajuda	4.) Quantas pessoas precisam de ajuda	4.) How many people need help.	4.) How many people need help
5.) My phone number	5.) Meu número de telefone	5. Meu número de telefone	5. Meu número de telefone	5. My own telephone number.	5. My telephone number
6.) Other: ________________________________	6.) Outros: ______________	6. Outro: [linha para preenchimento]	6. Outro: ______________	6. Something else:	6. Other information/details : ______________
3 What would you do if someone fell down in front of you and showed no signs of life? (more than one answer is possible)	03. O que você faria se alguém caísse na sua frente e não apresentasse sinais de vida? (mais de uma resposta é possível)	3. O que você faria se alguém caísse em sua frente e não mostrasse sinais de vida? (mais de uma resposta é p	3. O que você faria se alguém caísse na sua frente e não mostrasse sinais de vida? (mais de uma resposta é possível)	3. What would you do if someone fell over in front of you and showed no signs of life? (more than one reply is possible)	03. What would you do if someone fell down in front of you and showed no signs of life? (more
1.) I would walk away, I wouldn't dare go near that person	1. Eu me afastaria, não teria coragem de me aproximar da pessoa	1. Eu continuaria andando. Não teria coragem de me aproximar da pessoa.	1. Eu me afastaria, não teria coragem de me aproximar da pessoa	1. I'd move away from them; I wouldn't have the courage to get close to the person.	1. I would move away, I would be too afraid to approach the person
2.) I would call the emergency telephone number	2. Ligaria para o número de emergência	2. Eu chamaria o número de telefone de emergência	2. Eu ligaria para o número de emergência	2. I'd call the emergency telephone number.	2. I would call the emergency number
3.) I would check whether the person is conscious (is responsive)	3. Verificaria se a pessoa está consciente (apresenta reação)	3. Eu verificaria se a pessoa está consciente (se está reagindo)	3. Eu verificaria se a pessoa está consciente (se está reagindo)	3. I'd check whether the person was conscious (if they were reacting).	3. I would check to see if the person is conscious (if they are responding)
4.) If the person wasn't responsive, I would start providing CPR	4. Se a pessoa não reagir, eu começaria a aplicar a RCP	4. Se a pessoa não estiver reagindo, eu começaria a RCP	4. Se a pessoa não estiver reagindo, eu começaria a RCP	4. If the person was not reacting, I'd begin CPR.	4. If the person is not responding, I would start CPR
5.) If the person wasn't responsive, I would place them in the position for unconscious victims	5. Se a pessoa não reagir, eu a colocaria na posição indicada para vítimas que estão inconscientes.	5. Se a pessoa não estiver reagindo, eu a colocaria em uma posição para vítimas inconscientes	5. Se a pessoa não estiver reagindo, eu a colocaria na posição para vítimas inconscientes	5. If the person was not reacting, I'd place them in the position for unconscious victims.	5. If the person is not responding, I would put them in the position for unconscious victims
6.) Other: ________________________________	6. Outros: ____________________________	6. Outro: [linha para preenchimento]	6. Outro: ______________	6. Something else: ________	6. Other information/details :______________
4 What is the right position for unconscious victims (how do they have to be turned)?	04.Qual a posição correta para uma vítima inconsciente (como a vítima deve ser virada)?	4. Qual a posição correta para vítimas inconscientes (como elas devem ser viradas)?	4. Qual a posição correta para vítimas inconscientes (como elas devem ser viradas)?	4. What is the correct position for victims who are unconscious (how should they be turned)?	04. What is the correct position for unconscious victims (how should they be turned)?
1.) The right position is shown in picture A	1. A posição correta é mostrada na imagem A	1. A figura A é a posição correta	1. A figura A é a posição correta	1. Figure A is the correct position.	1. Figure A is the correct position
2.) The right position is shown in picture B	2. A posição correta é mostrada na imagem B	2. A figura B é a posição correta	2. A figura B é a posição correta	2. Figure B is the correct position.	2. Figure B is the correct position
3.) The right position is shown in picture C	3. A posição correta é mostrada na imagem C	3. A figura C é a posição correta	3. A figura C é a posição correta	3. Figure C is the correct position.	3. Figure C is the correct position
5 What is an automated external defibrillator (AED)? (please provide one answer)	05. O que é um desfibrilador externo automático (DEA)? (forneça uma resposta)	5. O que é um Desfibrilador Externo Automático (DEA)? (Assinale somente uma alternativa)	5. O que é um Desfibrilador Externo Automático (DEA)? (assinalar somente uma resposta)	5. What is an automated external defibrillator (AED)? (give only one reply)	05. What is an Automated External Defibrillator (AED)? (tick just one answer)
1.) An electronic device capable of recognizing if a person's heart is beating	1. Um dispositivo eletrônico capaz de verificar se o coração de uma pessoa está batendo.	1. É um aparelho eletrônico capaz de reconhecer se o coração de uma pessoa está batendo	1. Um dispositivo eletrônico capaz de verificar se o coração de uma pessoa está batendo.	1. An electronic device that can check if a person's heart is beating.	1. An electronic device than can check whether a person's heart is still beating.
2.) An electronic device that establishes a person's breathing	2. Um dispositivo eletrônico que verifica a respiração de uma pessoa.	2. É um aparelho eletrônico que restabelece a respiração de uma pessoa	2. Um dispositivo eletrônico que verifica a respiração de uma pessoa.	2. An electronic device that checks if a person is breathing.	2. An electronic device that checks a person's breathing.
3.) An electronic device that establishes the heart function	3. Um dispositivo eletrônico que verifica a função cardíaca.	3. É um aparelho eletrônico que restabelece a função cardíaca.	3. Um dispositivo eletrônico que verifica e restabelece a função cardíaca.	3. An electronic device that checks heart function and reestablishes it. [whether the heart rhythm is normal, and reestablishes	3. An electronic device that checks and restores heart function.
6 Does your school have an AED device?	06. Sua escola possui um dispositivo DEA?	6. Sua escola tem um aparelho DEA?	06. Sua escola tem um dispositivo DEA?	6. Does your school have an AED?	06. Does your school have an AED device?
1.) Yes	1. Sim	1. Sim	1. Sim	1. Yes.	1. Yes, it does
2.) No	2. Não	2. Não	2. Não	2. No.	2. No, it doesn't
3.) I don't know	3. Não sei	3. Não sei	3. Não sei	3. I don't know.	3. I don't know
7 Do you know of places or locations where an AED can be found?	07. Você sabe de algum lugar ou local onde é possível encontrar um DEA?	7. Você conhece lugares ou locais onde um DEA pode ser encontrado?	7. Você sabe onde encontrar um DEA?	7. Do you know where you can find an AED?	07. Do you know where to find an AED?
1.) Yes	1. Sim	1. Sim	1. Sim	1. Yes.	1. Yes, I do
2.) No	2. Não	2. Não	2. Não	2. No.	2. No, I don't
8 If the answer to the previous question was yes, please name some places or locations with AED devices:	08. Se a resposta anterior foi "sim", cite alguns lugares ou locais onde é possível encontrar um DEA:	8. Se sua resposta para a pergunta anterior foi sim, mencione alguns lugares ou locais com aparelhos DEA: [linh	08 Se a resposta anterior foi "sim", cite alguns lugares ou locais onde é possível encontrar um DEA:	8. If the previous reply was "yes", mention some of the places or locations where an AED can be found: ________	08. If the previous answer was "yes, I do", list some places or locations where it is possible to f
**SET 3: CPR KNOWLEDGE**	**CONJUNTO 3: CONHECIMENTOS SOBRE A RESSUSCITAÇÃO CARDIORESPIRATÓRIA (RCP)**	**PARTE 3: CONHECIMENTO SOBRE RCP**	**PARTE 2 : CONHECIMENTOS SOBRE A REANIMAÇÃO CARDIOPULMONAR (RCP)**	**PART 3 : KNOWLEDGE OF CARDIOPULMONARY RESUSCITATION (CPR)**	**PART 3: KNOWLEDGE ABOUT CARDIOPULMONARY RESUSCITATION (CPR)**
1 What do you first have to do at a scene with a person who is showing no signs of life or is unresponsive? (please provide one answer)	1 O que você deve fazer, em primeiro lugar, no local onde se encontra uma pessoa que não apresenta sinais de vida ou que não reage? (forneça uma resposta)	1. O que deve ser feito primeiro em uma situação onde uma pessoa não mostra sinais de vida ou não reage? (a	1. O que deve ser feito primeiro em uma situação onde uma pessoa não mostra sinais de vida ou não reage? (assinalar somente	1. What should be done first in a situation in which a person shows no signs of life, or is not reacting? (give only one reply)	01. What should be done first in a situation where a person shows no signs of life or is unresp
1.) Locate possible victims and secure the scene	1. Localizar possíveis vítimas e garantir a segurança do local	1. Localize possíveis vítimas e isole o local	1. Localizar possíveis vítimas e isolar o local	1. Locate other possible victims and isolate the location.	1. Locate possible victims and cordon off the area
2.) Dial 112 and wait for paramedics	2. Ligar para 112 e aguardar os socorristas	2. Disque 112 e espere pelos paramédicos	2. Ligar para 192 e aguardar o socorro	2. Call 192 and wait for help.	2. Call 192 and wait for help
3.) Secure the scene, locate possible victims and dial 112	3. Garantir a segurança do local, localizar possíveis vítimas e ligar para 112.	3. Isole o local, localize possíveis vítimas e disque 112	3. Isolar o local, localizar possíveis vítimas e ligar para 192.	3. Isolate the location, locate other possible victims and call 192.	3. Cordon off the area, locate possible victims and call 192.
2 How do you check if a person is breathing? (please provide one answer)	2 Como você verifica se uma pessoa está respirando (forneça uma resposta)	2. Como verificar se uma pessoa está respirando? (assinale somente uma resposta)	2. Como verificar se uma pessoa está respirando? (assinalar somente uma resposta)	2. How can I check if a person is breathing? (give only one reply)	02. How do you check if a person is breathing? (tick just one answer)
1.) Tilt the victim's head back, listen for breathing sounds over their mouth, and look to see if the chest is rising or falling	1. Incline a cabeça da vítima para trás, ouça os sons de respiração pela boca e observe se o peito está subindo e descendo.	1. Incline a cabeça da vítima para trás, ouça os sons de respiração pela boca e veja se o tórax está subindo ou c	1. Inclinar a cabeça da vítima para trás, ouvir os sons de respiração pela boca e observar se o tórax sobe e desce	1. Tilt the victim's head back, listen for sounds of breathing via the mouth, and observe whether the chest is rising and falling.	1. Tilt the victim's head back, listen for breathing sounds from their mouth, and see if their ches
2.) Tilt the victim's head back and look to see if the chest is rising or falling	2. Incline a cabeça da vítima para trás e observe se o peito está subindo e descendo.	2. Incline a cabeça da vítima para trás e veja se o tórax está subindo e descendo	2. Inclinar a cabeça da vítima para trás e observar se o tórax sobe e desce	2. Tilt the victim's head back and observe whether the chest is rising and falling.	2. Tilt the victim's head back and see if their chest is rising and falling
3.) Grab the victim by the shoulders and shake them	3. Segure a vítima pelos ombros, e dê uma chacoalhada..	3. Segure a vítima pelos ombros e sacuda	3. Segurar a vítima pelos ombros e sacudir	3. Hold the victim by the shoulders and shake them.	3. Hold the victim by the shoulders and shake
3 How deep should chest compressions be? (please provide one answer)	3 Qual deve ser a profundidade das compressões torácicas? (forneça uma resposta)	3. Quanto se deve comprimir o tórax? (assinale somente uma resposta)	3 Qual deve ser a profundidade das compressões torácicas? (assinalar somente uma resposta)	3. How deep should chest compressions be? (give only one reply)	03. How deep should the chest compressions be? (tick just one answer)
1.) 5 cm	1. 5 cm	1. 5 cm	1. 5 cm	1. 5 cm.	1. 5 cm.
2.) 7 cm	2. 7 cm	2. 7 cm	2. 7 cm	2. 7 cm.	2. 7 cm.
3.) 3 cm	3. 3 cm	3. 3 cm	3. 3 cm	3. 3 cm.	3. 3 cm.
4 How many chest compressions should be performed per minute? (please provide one answer)	4 Quantas compressões torácicas devem ser realizadas por minuto? (forneça uma resposta)	4. Quantas vezes por minuto se deve comprimir o tórax? (assinale somente uma resposta)	4 Quantas compressões torácicas devem ser realizadas por minuto? (assinalar somente uma resposta)	4. How many chest compressions should be given a minute? (give only one reply)	04. How many chest compressions should be performed a minute? (tick just one answer)
1.) 60 – 80 compressions per minute	1. 60-80 compressões por minuto	1. 60 – 80 compressões por minuto	1. 60 – 80 compressões por minuto	1. 60 – 80 compressions a minute.	1. 60 – 80 compressions a minute.
2.) 130 – 150 compressions per minute	2. 130-150 compressões por minuto	2. 130 – 150 compressões por minuto	2. 130 – 150 compressões por minuto	2. 130 – 150 compressions a minute.	2. 130 – 150 compressions a minute.
3.) 100 – 120 compressions per minute	3. 100-120 compressões por minuto	3. 100 – 120 compressões por minuto	3. 100 – 120 compressões por minuto	3. 100 – 120 compressions a minute.	3. 100 – 120 compressions a minute.
5 The image of a chest has different positions marked; please indicate the number of the position where hands must be placed when conducting chest compressions:	5 A imagem do peito tem várias posições marcadas; indique o número da posição onde as mãos devem ser colocadas para realizar as compressões torácicas:	5. A imagem do tórax tem diferentes posições marcadas; indique o número que corresponde à posição onde as	5. A imagem do tórax tem diferentes posições marcadas; indique o número que corresponde à posição onde as mãos devem ser	5. The chest image has different positions marked on it; indicate the number that corresponds to the position where the hands	05. The chest image has different positions marked; specify the number that corresponds to th
6 The image of a chest has different positions marked; please indicate the number of the position where AED electrodes must be placed: ______.	6 A imagem do peito tem várias posições marcadas; indique o número da posição onde os eletrodos do DEA devem ser colocados:	6. A imagem do tórax tem diferentes posições marcadas; indique o número que corresponde à posição onde os	6. A imagem do tórax tem diferentes posições marcadas; indique os números que correspondem às posições onde os eletrodos	6. The chest image has different positions marked on it; indicate the number that corresponds to the position where the AED	06. The chest image has different positions marked; specify the numbers that correspond to th
7 What is the correct procedure for using an AED? (more than one answer is possible)	7 Qual o procedimento correto para o uso do DEA? (mais de uma resposta é possível)	7. Qual o procedimento correto para utilizar o DEA? (mais de uma resposta é possível)	7. Qual o procedimento correto para utilizar o DEA? (mais de uma resposta é possível)	7. What is the correct procedure for using an AED? (more than one reply is possible)	07. What is the correct procedure for using an AED? (more than one answer is possible)
1.) Dial 112, stand by and wait for paramedics to arrive	1) Ligar para 112 e aguardar os socorristas.	1. Disque 112, permaneça no local e espere pela chegada dos paramédicos	1. Ligar para 192, permanecer no local e aguardar o socorro	1. Call 192, remain in the location and wait for help.	1. Call 192, remain at the scene and wait for help
2.) First check the victim's responsiveness and breathing, then switch on the AED and follow the voice instructions	2) Em primeiro lugar, verificar se a vítima está consciente e respirando e, então, ligar o DEA e seguir as instruções.	2. Verifique primeiro se a vítima está reagindo e respirando, então ligue o DEA e siga as instruções	2. Verificar primeiro se a vítima está reagindo e respirando, então ligar o DEA e seguir as instruções	2. First check if the victim is reacting and breathing, then switch on the AED and follow the instructions.	2. First check that the victim is reacting and breathing, then turn on the AED and follow the inst
3.) Dial 112 to get instructions on using the AED	3) Ligar para 112 para receber orientações sobre o uso do DEA	3. Disque 112 para instruções de como usar o DEA	3. Ligar para 192 para receber orientações sobre o uso do DEA	3. Call 192 to receive guidance on how to use the AED.	3. Call 192 to receive instructions regarding how to use the AED
8 How do you begin resuscitating a drowning victim? (please provide one answer)	8 Como você inicia a reanimação de uma vítima de afogamento? (forneça uma resposta)	8. Como começar a ressuscitar uma vítima de afogamento? (assinale somente uma resposta)	8 Como iniciar a reanimação de uma vítima de afogamento? (assinalar somente uma resposta)	8. How do you begin resuscitation with a victim of drowning? (give only one reply)	08. How to start resuscitation of a drowning victim? (tick just one answer)
1.) Check their breathing, start chest compressions and give them two breaths	1.) Verifique a respiração, inicie as compressões torácicas e faça duas respirações boca a boca.	1. Verifique a respiração, inicie a compressão torácica e faça duas respirações	1. Verificar a respiração, iniciar as compressões torácicas e realizar duas respirações boca a boca.	1. Check their breathing, start chest compression and give two mouth-to-mouth rescue breaths.	1. Check the person's breathing, start chest compressions and perform two rescue breaths.
2.) Check their breathing, give them five initial breaths, and then follow with chest compressions	2.) Verifique a respiração, faça cinco respirações boca a boca iniciais e, em seguida, faça as compressões torácicas.	2. Verifique a respiração, faça 5 respirações iniciais e continue com as compressões torácicas	2. Verificar a respiração, fazer cinco respirações boca a boca iniciais e, em seguida, fazer as compressões torácicas.	2. Check their breathing, give five initial mouth-to-mouth rescue breaths and then start chest compression.	2. Check the person's breathing, give five initial rescue breaths, then do chest compressions.
3.) Check their breathing and start conducting chest compressions	3.)Verifique a respiração e comece a realizar as compressões torácicas.	3. Verifique a respiração e inicie as compressões torácicas	3. Verificar a respiração e iniciar as compressões torácicas	3. Check their breathing and start chest compression.	3. Check the person's breathing and start chest compressions
**Set 4: Attitudes towards a person needing help**	**Conjunto 4: Atitudes em relação a uma pessoa que precisa de ajuda**	**Parte 4: Atitudes em relação a uma pessoa que precisa de ajuda**	**Parte 4: Atitudes em relação a uma pessoa que precisa de ajuda**	**Set 4: Attitudes towards a person needing help**	**Set 4: Attitudes towards a person needing help**
1. If someone fell down in front of me, I would help them.	1. Se alguém caísse na minha frente, eu o ajudaria.	1. Se alguém caísse na minha frente, eu o ajudaria.	1. Se alguém caísse na minha frente, eu o ajudaria.	1. If someone were to fall in my presence, I would help them.	1. If someone fell down in front of me, I would help them.
2. If my friend lost consciousness, I would help them.	2. Se o meu amigo ficasse inconsciente, eu o ajudaria.	2. Se meu amigo perdesse a consciência, eu o ajudaria.	2. Se meu amigo perdesse a consciência, eu o ajudaria.	2. If a friend of mine were to lose consciousness, I would help them.	2. If my friend lost consciousness, I would help him or her.
3. I am willing to help others because I would also expect help when in need.	3. Estou disposto a ajudar os outros porque também espero ser ajudado quando precisar.	3. Procuro ajudar os outros porque também espero ter ajuda quando precisar.	3. Procuro ajudar os outros porque também espero ter ajuda quando precisar.	3. I try to help others because I hope I'll receive help when I need it.	3. I try to help others because I would also hope they would help if I needed it
4. I help because I can recognize a person who is not showing signs of life.	4. Ajudo porque sei reconhecer uma pessoa que não apresenta sinais de vida.	4. Eu ajudo porque consigo reconhecer quando uma pessoa não está mostrando sinais de vida.	4.Ajudo porque sei reconhecer quando uma pessoa não está mostrando sinais de vida.	4. I help because I can recognize when someone is not showing signs of life.	4. I help because I can recognize when a person is not showing any signs of life.
5. I like to help others when they are in need.	5. Gosto de ajudar os outros quando precisam de ajuda.	5. Gosto de ajudar os outros quando precisam.	5. Gosto de ajudar os outros quando precisam.	5. I like helping others when they need it.	5. I like to help others when they need it.
6. I notice when my friends and classmates need help.	6. Percebo quando meus amigos e colegas de classe precisam de ajuda.	6. Percebo quando meus amigos e colegas de classe precisam de ajuda.	6. Percebo quando meus amigos e colegas de classe precisam de ajuda.	6. I notice when my friends and classmates need help.	6. I notice when my friends and classmates need help.
7. When I notice that someone needs help, I ask myself how I can help them.	7. Quando percebo que alguém precisa de ajuda, pergunto a mim mesmo como posso ajudá-lo.	7. Quando percebo que alguém precisa de ajuda, pergunto a mim mesmo como posso ajudar.	7. Quando percebo que alguém precisa de ajuda, pergunto a mim mesmo como posso ajudar.	7. When I notice that someone needs help, I ask myself how I can be of assistance.	7. When I notice that someone needs help, I ask myself how I can help.
8. If someone fell down in front of me, I would call for help immediately.	8.Se alguém caísse na minha frente, eu pediria ajuda imediatamente.	8. Se alguém caísse na minha frente, eu pediria ajuda imediatamente.	8. Se alguém caísse na minha frente, eu pediria ajuda imediatamente.	8. If someone were to fall in my presence, I'd immediately call for help.	8. If someone fell down in front of me, I would immediately call for help.
9. If I noticed a group of people only observing a victim not showing signs of life, I would start providing help immediately.	9. Se eu notasse um grupo de pessoas apenas observando uma vítima sem sinais de vida, eu começaria a ajudar imediatamente.	9. Se eu notasse um grupo de pessoas apenas observando uma vítima que não estivesse dando sinais de vida,	9. Se eu notasse um grupo de pessoas apenas observando uma vítima sem sinais de vida, eu começaria a prestar ajuda imediat	9. If I saw a group of people just observing a person with no signs of life, I'd begin to help immediately.	9. If I noticed a group of people just watching a victim with no signs of life, I would immediately
10. I am aware that by helping a victim not showing signs of life, I can save their life.	10.Tenho consciência de que, ao ajudar uma vítima sem sinais de vida, posso salvar sua vida.	10. Estou ciente de que, ao ajudar uma vítima que não está mostrando sinais de vida, posso salvar sua vida.	10. Sei que, ao ajudar uma vítima sem sinais de vida, posso salvar sua vida.	10. I know that when I'm helping a person with no signs of life I can save their life.	10. I know that by helping a victim who is not showing any signs of life, I may be able to save h
11. If someone fell down in front of me and showed no signs of life, I would start CPR.	11. Se alguém caísse na minha frente e não apresentasse sinais de vida, eu iniciaria a RCP.	11. Se alguém caísse na minha frente e não mostrasse sinais de vida, eu iniciaria a RCP.	11. Se alguém caísse na minha frente sem sinais de vida, eu iniciaria a RCP.	11. If someone were to fall in my presence with no signs of life, I'd start CPR.	11. If someone fell down in front of me without any signs of life, I would start CPR.
12. If I noticed a victim who was not moving or showing signs of life, I would begin immediately, because I am not afraid of injuring them.	12.Se eu visse uma vítima que não estivesse se mexendo ou mostrando sinais de vida, eu iniciaria o atendimento imediatamente, porque não tenho medo de machucá-la.	12. Se eu percebesse que uma vítima não estivesse se movendo ou mostrando sinais de vida, eu começaria ime	12.Se eu notasse uma vítima que não estivesse se movendo ou sem sinais de vida, eu começaria o atendimento imediatamente,	12. If I noticed a person not moving or showing any signs of life, I'd begin helping them immediately, because I'm not afraid of	12. If I noticed a victim who was not moving or showing any signs of life, I would immediately s
13. If I noticed a victim who was not moving or showing signs of life, I would begin CPR immediately because I believe in myself.	13. Se eu visse uma vítima que não estivesse se mexendo ou mostrando sinais de vida, eu iniciaria a RCP imediatamente, pois confio em mim.	13. Se eu notasse que uma vítima não estivesse se movendo ou mostrando sinais de vida, eu iniciaria a RCP im	13. Se eu notasse uma vítima que não estivesse se movendo ou sem sinais de vida, eu iniciaria a RCP imediatamente, porque c	13. If I noticed a person was not moving or showing any signs of life, I'd start CPR immediately, because I trust myself.	13. If I noticed a victim who was not moving or showing any signs of life, I would immediately s
14. When friends ask for my help, I don't hesitate and help them right away.	14. Quando amigos me pedem ajuda, não hesito e os ajudo imediatamente.	14. Quando amigos pedem minha ajuda, eu não hesito e os ajudo imediatamente.	14. Quando amigos pedem minha ajuda, eu não hesito e os ajudo imediatamente.	14. When friends ask me to help them I don't hesitate; I help immediately.	14. When friends ask for my help, I do not hesitate and help them immediately.
15. I feel for classmates and friends who are very ill or were struck by misfortune.	15. Sinto empatia por colegas de classe e amigos que estão muito doentes ou que passaram por algum infortúnio.	15. Sinto pena dos colegas de classe e amigos que estão muito doentes ou foram atingidos por um infortúnio.	15. Sinto pena dos colegas de classe e amigos que estão muito doentes ou foram atingidos por um problema sério.	15. I feel sorry for classmates and friends who are very sick, or have suffered a serious problem.	15. I feel sorry for classmates and friends who are very sick or have been affected by a serious
16. I think one of the best things is being able to help others.	16. Acredito poder ajudar os outros é ótimo.	16. Acho que uma das melhores coisas é poder ajudar os outros.	16. Acho que uma das melhores coisas é poder ajudar os outros.	16. I think that being able to help others is one of the best things there is.	16. I think that being able to help others is one of the best things of all.
17. Helping others gives me satisfaction.	17. Sinto satisfação em ajudar os outros.	17. Sinto satisfação em ajudar os outros.	17. Sinto satisfação em ajudar os outros.	17. I get satisfaction from being able to help others.	17. I get satisfaction from helping others.
18. I would dare to provide CPR before receiving training.	18. Eu me arriscaria a realizar RCP antes de receber treinamento.	18. Eu teria coragem de fazer RCP antes de receber treinamento.	18. Eu teria coragem de fazer RCP antes de receber treinamento.	18. I'd have the courage to administer CPR before receiving any training.	18. I would have the courage to perform CPR before receiving training.
19. I would dare to provide CPR after receiving training.	19. Eu me arriscaria a realizar RCP após receber treinamento.	19. Eu teria coragem de fazer RCP depois de receber treinamento.	19. Eu teria coragem de fazer RCP depois de receber treinamento.	19. I'd have the courage to administer CPR after receiving training.	19. I would have the courage to perform CPR after receiving training.
1 - Strongly disagree	1- Discordo totalmente	1- Discordo totalmente	1- Discordo totalmente	1 - Strongly disagree	1 - Strongly disagree
2 - Disagree	2- Discordo	2- Discordo	2- Discordo	2 - Disagree	2 - Disagree
3 - Neither agree nor disagree (undecided)	3- Nem concordo nem discordo	3- Nem concordo nem discordo	3- Nem concordo nem discordo	3 - Neither agree nor disagree (undecided)	3 - Neither agree nor disagree (undecided)
4 - Agree	4- Concordo	4- Concordo	4- Concordo	4 - Agree	4 - Agree
5 - Strongly agree	5. Concordo totalmente	5. Concordo totalmente	5. Concordo totalmente	5 - Strongly agree	5 - Strongly agree
